# Characteristics Verification of an Independently Controllable Electromagnetic Spherical Motor

**DOI:** 10.3390/s140610072

**Published:** 2014-06-10

**Authors:** Shuhei Maeda, Katsuhiro Hirata, Noboru Niguchi

**Affiliations:** Department of Adaptive Machine Systems, Graduate School of Engineering, Osaka University, 2-1 Yamadaoka Suita, Osaka 565-0871, Japan; E-Mails: k-hirata@ams.eng.osaka-u.ac.jp (K.H.); noboru.niguchi@ams.eng.osaka-u.ac.jp (N.N.)

**Keywords:** actuator, electromagnetic analysis, finite element method, permanent magnet motors

## Abstract

We have been developing electromagnetic spherical actuators capable of three-degree-of-freedom rotation. However, these actuators require complex control to realize simultaneous triaxial drive, because rotation around one axis interferes with rotation around another. In this paper, we propose a new three-degree-of-freedom actuator where 3-axes rotation can be controlled easily. The basic structure and the operating principle of the actuator are described. Then the torque characteristics and the dynamic characteristics are computed by employing 3D-FEM and the effectiveness of this actuator is clarified. Finally, the experimental results using the prototype of the actuator are shown to verify the dynamic performance.

## 1. Introduction

Conventionally, a multi-degree-of-freedom drive mechanism such as a robot arm is typically composed of more than one motor. However, this mechanism has some problems including a deterioration in efficiency, and an increase in weight and size. Therefore, multi-degree-of-freedom actuators are expected to become a key technology for downsizing and increasing the efficiency of the mechanism, and thus many types of actuators have been studied [1,2]. Especially, spherical actuators can rotate three dimensionally and are being extensively studied since they can be applied to the joints and eyeballs of robots [3–8]. While spherical actuators have many advantages, there are some outstanding challenges. Current problems of spherical actuator design include low torque, narrow rotation angle, low-precision positioning, sensing method, mover support *etc*.

We have been studying various kinds of spherical actuators [9,10] and the torque characteristics of the actuators were analyzed using the 3-D Finite Element Method (FEM). Additionally, we have been developing the control method and position sensing system for the spherical actuators using optical image sensors [11].

However, independent control of the three axes has not been achieved. In this paper, we propose a new three-degree-of-freedom actuator where 3-axes rotation can be controlled easier than the previous 3-axes rotational motors. First, the basic structure and the operating principle of the actuator are described. Then the torque characteristics and the dynamic characteristics are computed employing 3D-FEM and the effectiveness of this actuator is clarified. Finally, the experimental results using the prototype of the actuator are shown to verify the dynamic performance.

## 2. Description of the Actuator

**Figure 1.**
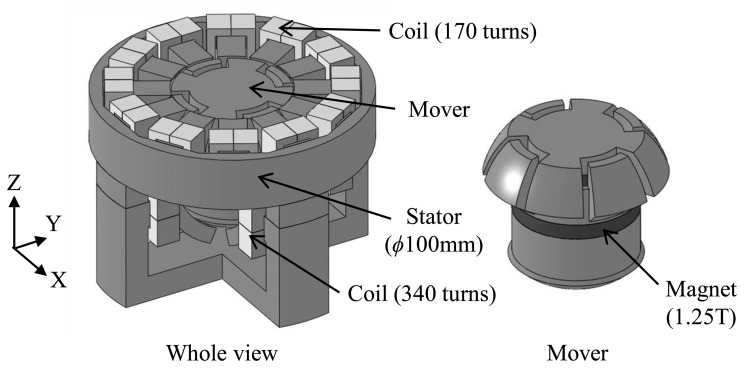
Basic structure of the actuator: whole model (**Left**) and enlarged view of the mover (**Right**).

**Figure 2.**
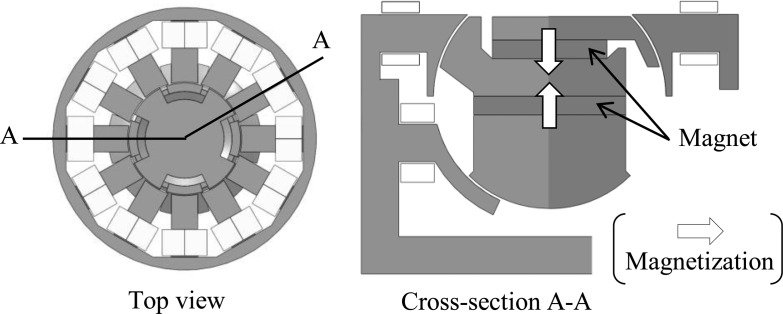
Cross-section view of the actuator.

The proposed actuator is designed for the realization of triaxial simultaneous rotation by separating the tilt and rotation drive mechanism. The basic structure of this actuator and its cross-section view are represented in Figures 1 and 2. This actuator is mainly composed of an inner mover and an outer stator. The stator is divided further into an upper half for rotation drive and a lower half for tilt drive. Each stator pole has a coil wound around it (upper 170 turns, lower 340 turns respectively). The mover is composed of two permanent magnets (Br = 1.25 T) and magnetic bodies as shown in Figure 2. The air gap between the mover and the stator is kept constant (0.7 mm) during motion. The outer diameter and the height of the stator are 100 mm and 70 mm respectively.

**Figure 3.**
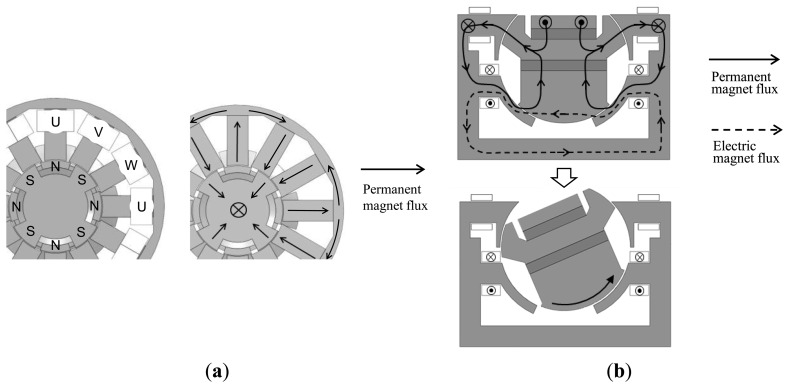
Operating principle of the actuator: (**a**) rotation drive; (**b**) tilt drive.

In this section, the operating principle of the actuator is illustrated. During rotation drive, the coils in the upper half of the stator are used to generate a rotating magnetic field when excited by a three-phase AC supply. As shown in Figure 3a, north and south magnetic poles appear alternately on the upper teeth of the mover, and thus the mover rotates as a synchronous motor.

On the other hand, the coils in the lower half of the stator are used during tilt drive to strengthen or weaken the flux from the permanent magnets. In the example shown in Figure 3b, the coils weaken the flux from the permanent magnets on the left and strengthen on the right. This causes an imbalance in the symmetry of the flux distribution in the proposed actuator and as a result, the mover rotates in the direction of the arrow. The mover rotates in the counter direction when the coils are conversely excited, and the operating principle of the X-axis and Y-axis is the same.

The bottom of the mover is axisymmetric, so rotation drive has only a very small effect on tilt drive. This actuator can be three-dimensionally rotated by combining these operating methods and therefore the common inverter is available for the actuator.

Although it may seem that by separating the drive mechanism, we are just merely joining two motors together, the novelty of our design is that the magnetic flux generated by the permanent magnets is shared between the upper and lower halves of the stator, and this allows for the shape of the magnets to become simpler and the actuator to become smaller.

## 3. Characteristics Analysis

An electromagnetic field analysis using 3-D FEM is conducted to determine the static torque characteristics and the dynamic operating characteristics of the proposed actuator.

### 3.1. Torque Characteristics Analysis

**Figure 4.**
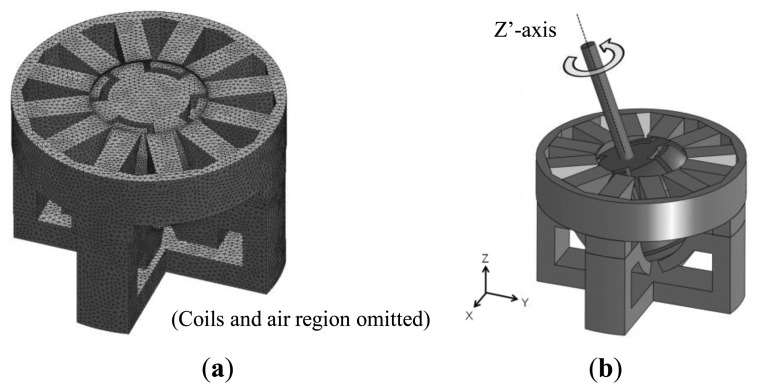
Analysis model of the actuator: (**a**) 3D mesh model; (**b**) Simultaneous rotation and tilt drive.

Static torque characteristic in multi-axial rotation was conducted to see how rotation around one axis affects rotation around another. The 3-D finite element mesh model used in the analyses and the operating condition are shown in Figure 4. The mover was rotated around the Z′ axis, which is the Z axis that has been rotated 15 degrees around the X axis.

**Figure 5.**
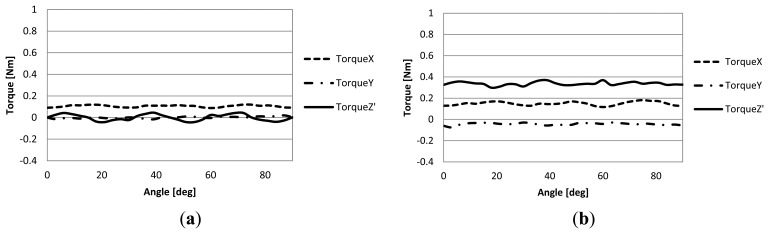
Torque characteristics analysis: (**a**) Cogging torque; (**b**) Output torque.

Each lower pole is excited by 1A DC for tilt drive, and the upper poles are excited by 1A 3-phase AC during rotation drive. In addition to the output torque characteristics, the cogging torque characteristics are also computed in each analysis. The results of the analyses are shown in Figure 5. The horizontal axis of each graph shows the rotation angle around the Z′ axis.

From the analyzed result of the cogging torque, it can be seen that little cogging torque is generated around the Y-axis and the Z-axis, but the torque around the X-axis is constant at about 0.1 Nm since the mover is tilted. This is the force that is trying to rotate the mover back to its position of equilibrium. As for the output torque, the Z′-axis torque is about 0.34 Nm.

The important information obtained from these results is that the output torque was always positive in all of the analyses. This shows that the actuator can rotate in the analyzed range. In addition to this, a notable characteristic is that the output torque around the X-axis during simultaneous rotation and tilt drive (dashed line in Figure 5b) is relatively flat and has little ripple. If there is a noticeable ripple, the current for tilt drive must be adjusted constantly to maintain the tilt angle. Therefore, it can be deduced that simultaneous three degrees of freedom control can be easily accomplished and tilt drive and rotation drive can be controlled independently.

### 3.2. Operating Characteristics Analysis

**Figure 6.**
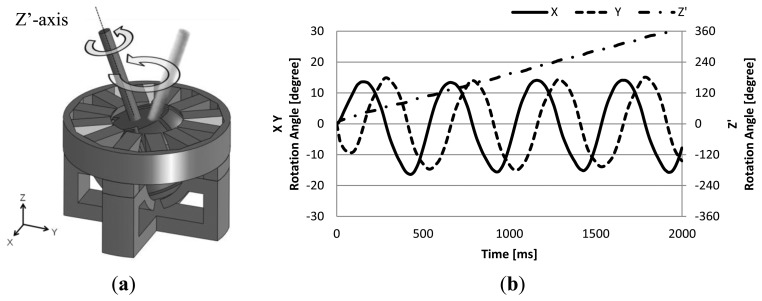
Open-loop control analysis: (**a**) Motion of the output shaft during triaxial drive; (**b**) Analysis result.

Dynamic operating characteristic analyses were conducted to confirm how the mover behaves under open-loop control and position feedback control. In open-loop control analysis, the actuator rotates around 3 axis simultaneously as shown in Figure 6a. The lower magnetic poles are excited by a 1.5 A, 2 Hz AC current for tilt drive, and the upper twelve magnetic poles are excited by a 1.5 A, 2 Hz three-phase AC for rotation drive.

The analyzed result during triaxial simultaneous drive under open-loop control is shown in Figure 6b. From the analyzed result, it can be confirmed that the mover is rotating around the Z′-axis, and at the same time the Z′-axis is rotating along a circular path. This result shows that simultaneous triaxial drive is possible by just simply combining the current control of each uniaxial drive.

**Figure 7.**
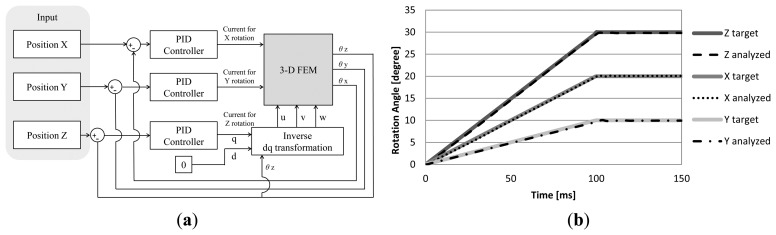
Closed-loop control analysis: (**a**) block diagram of the position feedback control; (**b**) analysis result.

In closed-loop control analysis, the mover is controlled by PI control, and the block diagram of the control is shown in Figure 7a. The target angles of the X, Y and Z axis at 100 milliseconds are 20, 10 and 30 degrees, respectively. Figure 7b shows the operating characteristics during triaxial simultaneous drive under position feedback control. This result also shows that the position feedback control was successful around all axes, and that the proposed actuator can simultaneously rotate around 3-axes accurately with this simple control method.

## 4. Experimental Verification

In the previous section, it was confirmed that the proposed actuator can rotate with three dimensional independent control. In this section, the results of the experiment conducted to verify the analysis results are shown.

### 4.1. Experimental Method

**Figure 8.**
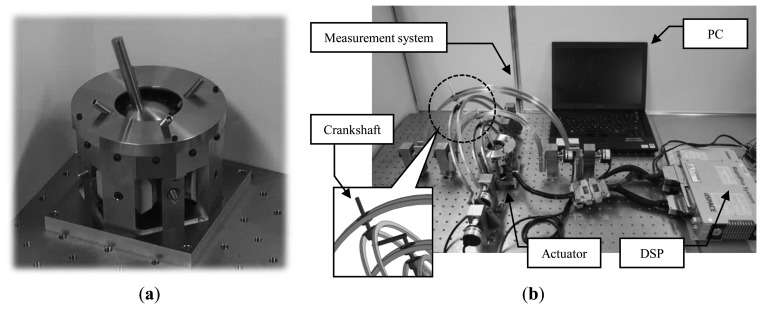
Verification experiment: (**a**) prototype of the actuator; (**b**) experiment system.

The prototype of the proposed actuator used in the experiment and the experimental equipment are shown in Figure 8. To control of the actuator, a PC and Digital Signal Processor (DSP) are employed. The measurement equipment is composed of four arch-shaped parts and four rotary encoders and a crankshaft. It can measure the angle of the spherical actuator by converting the outputs of four encoders into three dimensional angle.

In the experiment under open-loop control, the motion pattern and the inputs to each coil are the same as that of the analysis condition. In the experiment under closed-loop control, the measured angle of the mover is input into the DSP and is used for the angle feedback control.

### 4.2. Experimental Results

**Figure 9.**
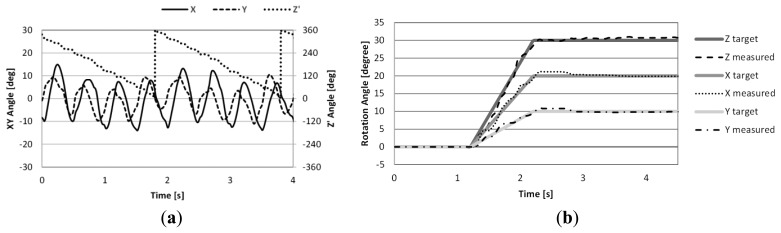
Experiment results: (**a**) rotation angle of the mover during open-loop control; and (**b**) closed-loop control.

The experimental results of open-loop control drive are shown in Figure 9a. From the results, the reciprocating motion around the X and Y axes and the rotary motion around the Z′ axis can be observed. However the amplitude of the motion around the X and Y axes are small and unstable compared to the analysis result. This is attributed to the frictional force which is not considered in the numerical analyses.

The experimental results under closed-loop control are shown in Figure 9b. The position feedback functioned properly and the final angles of each axis were 19.89°, 9.9° and 30.7°, respectively. The errors are within one degree. However unstable behavior can be observed during operation as was also the case in open-loop control, and the maximum error of each axis were 1.63°, 1.44° and 2.32°, respectively. From these experimental results, it can be confirmed that the proposed actuator can rotate three dimensionally following the target angle under position feedback control even though there is room for improvement in the control method.

## 5. Conclusions

In this paper, a new electromagnetic spherical actuator was proposed to overcome the shortcomings of traditional actuators on complex triaxial control. The basic structure and operating principle of the proposed actuator were presented, and its torque characteristics and operating characteristics were investigated through electromagnetic field analysis using 3D-FEM and experimental verification.

The analysis results and the experimental results show that the proposed actuator can be independently controlled to simultaneously rotate around the three axes and the actuator can rotate under position feedback control which is applied to each axis independently. The challenges we plan to tackle in the future of this study are the development of a high-torque spherical actuator and the absolute position sensing method for spherical actuators.
